# The feasibility and usability of a personal health record for patients with multiple sclerosis: a 2-year evaluation study

**DOI:** 10.3389/fnhum.2024.1379780

**Published:** 2024-05-22

**Authors:** Liselot N. van den Berg, Jiska J. Aardoom, Léone E. Kiveron, Robert D. Botterweg, M. Elske van den Akker – van Marle, Niels H. Chavannes, Elske Hoitsma

**Affiliations:** ^1^Department of Public Health and Primary Care, Leiden University Medical Center, Leiden, Netherlands; ^2^National eHealth Living Lab, Leiden, Netherlands; ^3^Department of Neurology, Alrijne Hospital Leiden, Leiden, Netherlands; ^4^Department of Biomedical Data Sciences, Medical Decision Making, Leiden University Medical Center, Leiden, Netherlands

**Keywords:** multiple sclerosis, MS, implementation, personal health record, eHealth, health communication, feasibility, usability

## Abstract

**Background:**

Multiple sclerosis (MS) is a persistent inflammatory condition impacting the brain and spinal cord, affecting globally approximately 2.8 million individuals. Effective self-management plays a crucial role in the treatment of chronic diseases, including MS, significantly influencing health outcomes. A personal health record (PHR) is a promising tool to support self-management, potentially empowering patients and enhancing their engagement in treatment and health. Despite these promising aspects, challenges in implementation persist and PHRs are still a relatively new concept undergoing rapid development.

**Objective:**

This study aimed to assess the feasibility and usability of the PHR. Secondary objectives included evaluating implementation determinants, and exploring preliminary effects on quality of care for both patients and healthcare professionals (HCPs), self-management, self-efficacy for patients, job satisfaction, efficiency, and demand for HCPs, and preliminary effects on costs and health-related quality of life.

**Methods:**

This study had a mixed-methods design. Quantitative data of patients (*n* = 80) and HCPs (*n* = 12) were collected via self-reported questionnaires at baseline (T0), after one year (T1), and after two years (T2). One focus group interview was conducted at T2 with patients (*n* = 7), and another one with HCPs (*n =* 4), to get a more in-depth understanding of the feasibility and usability of the PHR via the Unified Theory of Acceptance and Use of Technology framework, and to further explore the secondary objectives in-depth.

**Results:**

Most patients never logged in during the first year and logged in a couple of times per year during the second year, averaging around 15 min per log-in session. The HCPs mainly logged in a couple of times per year over the two years with an average use of six minutes per session. Patient usability and satisfaction scores were below average and moderate, respectively: with SUS-scores of 59.9 (*SD* = 14.2, *n* = 33) at T1 and 59.0 (*SD* = 16.3, *n* = 37) at T2, and CSQ-8 scores of 21.4 (*SD* = 5.0, *n* = 34) at T1, and 22.1 (*SD* = 5.0, *n* = 39) at T2. HCPs had similar usability and satisfaction scores. Multiple facilitators and barriers were identified by both patients and HCPs, such as (in)sufficient knowledge of how to use the PHR, lack of staff capacity and ICT obstacles. No significant differences were found in the preliminary effects. Qualitative data showed, among others, that both patients and HCPs saw the benefit of the PHR in terms of performance expectancy, by gaining more insight into health and health data, but challenges remained regarding effort expectancy, such as log-in issues and experiencing difficulties with information retrieval.

**Conclusion:**

The feasibility and usability were considered moderate by patients and HCPs; however, potential regarding the performance of the PHR was observed. Implementation challenges, such as the complexity of usage, lowered the adoption of the PHR. The evolving nature of PHRs requires ongoing evaluation and adaptation to optimize their potential benefits. Utilizing a participatory design approach and a dedicated implementation team could help in achieving this optimization, ultimately enhancing their adoption.

## Introduction

Multiple sclerosis (MS) is a chronic inflammatory condition affecting the brain and spinal cord, damaging myelin and axons in the white and grey matter (Compston and Coles, 2008; Ghasemi et al., 2017). The early course of MS is characterized by episodes of neurological dysfunction, generally followed by periods of remission (Compston and Coles, 2008). Over time, MS often results in physical and cognitive impairments, and an increase in neurological problems (Ghasemi et al., 2017), with symptoms such as fatigue, vision problems, numbness, difficulties with balance and coordination, and problems with thinking, learning and planning (NHS, 2023). The first symptoms of MS often occur between the 20th and 40th year of life (Poser et al., 1982). It is more common in women than men (NHS, 2023). Around 2.8 million individuals live with MS globally (Walton et al., 2020) and the disease burden in terms of Disability-Adjusted-Life-Years (DALYs) is substantial (Collaborators GBDMS, 2019). A multidisciplinary approach is needed to manage all MS symptoms (Feys et al., 2016), and costs for patients with MS are expected to increase over time when the disease worsens and becomes more severe (Kobelt et al., 2006).

Besides medication, different types of therapies (e.g., physiotherapy, exercise therapy and speech therapy) can have a positive impact on alleviating symptoms. These therapies may help people with MS by empowering them and increasing their self-management (Rae-Grant et al., 2011). Self-management is an important aspect in the treatment of chronic diseases, including MS, due to its significant impact on health outcomes (Lorig and Holman, 2003; McGowan, 2012; Grady and Gough, 2014). For instance, self-management can enhance individuals’ quality of life (QoL) and coping with the disease (Novak et al., 2013). Many patients with MS prefer to play an active role in managing MS to maintain functioning in their daily lives (Knaster et al., 2011). Individuals with chronic diseases have previously experienced success with eHealth tools and interventions which enhance self-management (Lorig et al., 2006; Elbert et al., 2014). For patients with MS, eHealth may help to improve self-management by, for example, tracking adherence to treatment and their psychological well-being. It can also support healthcare professionals (HCPs) by enabling them to remotely monitor the patient’s symptoms and share clinical data with their patients (Marziniak et al., 2018). Other factors that are important in the treatment of MS are proactive monitoring, (early) treatment access and shared decision-making processes (Giovannoni, 2016). A personal health record (PHR) could be a tool to support self-management and enhance care for patients with MS.

A PHR is an individually maintained electronic record designed for monitoring and overseeing one’s personal health information within a secure data environment (Spil and Klein, 2015). On a multifunctional platform, patients and HCPs can exchange information (Roehrs et al., 2017), and a PHR can consist of different features, such as administrative features (e.g., booking consultations tool) and clinical features (e.g., reviewing subjective experience data, for example, measured QoL and objective data, for example, radiology data and blood tests results) (Price et al., 2015). Additionally, a PHR often allows patients and HCPs to communicate with each other. Some PHRs allow patients to receive disease-specific reminders (e.g., to take medication) which support self-management (Price et al., 2015). PHRs differ from electronic patient portals because they are owned and administrated by patients themselves, instead of by healthcare institutions, and have more advanced features than only being able to access one’s personal health information (Ammenwerth et al., 2012). The goal of a PHR is to increase empowerment and improve the patient’s engagement in their treatment and health (Price et al., 2015). A PHR can have multiple advantages, such as decreasing the communication barrier between patients and HCPs, and improving the shared decision-making process, which can result in better quality of care (Aslani et al., 2020). Furthermore, a PHR may also have potential societal benefits, such as lower chronic disease management costs (Tang et al., 2006).

However, implementation challenges remain, such as poor adoption rates and poor integration into care processes (Archer et al., 2011). Although this might partly be explained by the fact that PHRs are relatively new within the landscape of eHealth, the literature also shows that there are negative experiences with user-friendliness, especially for users with limited digital health literacy, and concerns about privacy and security (Archer et al., 2011; Kruse et al., 2015). Moreover, HCPs may experience increased pressure when they receive more messages that require prompt responses, and they may experience a lack of guidance when there is no proper training to learn how to work with a PHR in their daily healthcare practices (Kruse et al., 2015). The question also remains how the funding of PHRs will be realised in the future (Archer et al., 2011; Kruse et al., 2015).

A PHR for patients with MS has been implemented in a Dutch hospital as a standard component of MS patient care. In the Netherlands, every Dutch person has the legal right to electronic access and copies of medical files from their HCP since July 1 2020. In this context, the PHR (in Dutch: ‘*Persoonlijke GezondheidsOmgeving*) was developed. PHRs are therefore a relatively new concept in the Netherlands still undergoing rapid changes. Previous studies underlined the necessary follow-up research on PHRs and their impact on patients, HCPs and the healthcare system (Archer et al., 2011; Price et al., 2015). Therefore, the primary aim of this study was to evaluate the feasibility and usability of the PHR within the hospital. The secondary aims were to (a) assess the implementation determinants related to the usage and evaluation of the PHR, and (b) examine the preliminary effects of using the PHR on (b.1) the experienced quality of care from both the perspective of the patients and HCPs, (b.2) self-management and self-efficacy of patients, (b.3) job satisfaction, job efficiency and job demand of HCPs, and (b.4) preliminary effects on the costs and health-related QoL.

## Methods

### Study design and population

This study had a mixed-methods design. Both patients with MS from the *Alrijne* Hospital (Leiden, the Netherlands) and their respective HCPs, who treated these patients, participated in this study. The inclusion criteria were only applicable to the patient population. Individuals were eligible to participate when they were (a) aged 18 years or older and (b) proficient in understanding and speaking Dutch, allowing them to comprehend the PHR. The study period consisted of two years with three quantitative measurements: at baseline; before the implementation of the PHR (T0), and one (T1) and two years after the implementation of the PHR (T2), respectively. Additionally, at T2, focus group interviews were organized: one focus group with patients and one focus group with HCPs. The study ran from October 2020 to February 2023. Recruitment took place between September 2020 and January 2021.

This study did not fall within the scope of the Dutch Medical Research Involving Human Subjects Act, according to the Medical Ethics Committee of Leiden University Medical Center (LUMC; N20.079).

### The PHR

The PHR was set up to gain better overview of the disease course, including both in-hospital gathered data and Patient-Reported Outcome Measures (PROMs), and to enhance self-management. The PHR was set up in cooperation with *MedMij, Enovation* (Capelle aan den IJssel, the Netherlands) and *CuraVista* (Raamsdonksveer, the Netherlands) to enhance standardized and secure data exchange. *MedMij* is a program initiated by the Ministry of Health, Welfare and Sport, where parties make agreements about a national ICT infrastructure and data exchange. ICT company *Curavista* developed the PHR. *Enovation* provided the connection between the hospital information system and the PHR.

From the hospital information system, data relating to Magnetic Resonance Imaging (MRI) test results, blood test results such as JC virus screening, clinimetry test results (see Supplementary Figure S1; e.g., six minute walk test, 25 foot walk test, nine hole PEG test, Berg Balance score, timed up and go score), course of MS (Expanded Disability Status Scale), medical treatments, and clinical disease activity (e.g., relapses), are exchanged within the PHR. Furthermore, once per year a goal for the next year, such as smoking cessation, walking for 30 min per day or implementing a resting moment per day, was set in the PHR by both the patients and the HCPs, in order to gain the same goal and a positive mindset by both parties.

From the PHR, PROMs such as QoL [EQ-5D (EuroQoL Research Foundation, 2019)] and MS-specific questionnaires related to the disease course [the Multiple Sclerosis Impact Profile (MSIP; Wynia et al., 2008), see Supplementary Figure S2] and bladder function [including the Actionable bladder system screening questionnaire (Burks et al., 2013; Jongen et al., 2015)] are exchanged with the hospital information system. Both sets of data are displayed in a dashboard in a comprehensible manner (see Supplementary Figures S1, S2).

Patients are also able to enter self-help functionalities, such as a digital decision aid “Do I have a relapse?,” a rest diary that might help to gain insights in the effect of rest on QoL, and the “Uricontrol-module,” which offers bladder training for an overactive bladder and pelvic floor muscle exercises for weaking of the pelvic floor muscles (see Supplementary Figure S3).

As we wanted to test feasibility and usability in daily practice without personal support we decided not to give personal assistance on how to use the PHR. Patients just received a small letter with user instructions.

### Procedure

Recruitment of this study took place via an invitation letter with the informed consent form, an expression of interest form, instructions for the PHR, and a reply envelope which was signed by the neurologist who initiated the study (EH). This neurologist was not a subject in the study. These materials were also distributed within the hospital to recruit HCPs (i.e., physical therapists, urologists, rehabilitation doctors, neurologists, and MS nurses), specifically to the departments associated with MS care and where the PHR would be implemented. On the expression of interest form, interested patients and HCPs could provide their gender, name, telephone number and email address. Researcher LvdB subsequently contacted them via telephone to confirm their interest in participating in the study, to inquire if they had any questions about the study, and to check their eligibility (in the case of the patients). Following this, eligible individuals could sign the informed consent form and send it back to the LUMC. In the informed consent form, both patients and HCPs received a question about whether they would be interested in participating in a focus group interview.

Afterwards, patients received a link to the Castor database (i.e., a digital, secure research environment) (Castor, 2023), in which they could complete the socio-demographical and clinical characteristics questionnaire and other questionnaires about quality of care, self-management [Partners in Health scale (PIH; Battersby et al., 2003)], self-efficacy [Multiple Sclerosis Self-Efficacy Scale (MSSE; Schwartz et al., 1996)], healthcare use [iMTA Medical Consumption Questionnaire (iMCQ; Erasmus University Rotterdam, 2023)], health-related QoL [EQ-5D-5L; (EuroQoL Research Foundation, 2019) consisting of a descriptive system of five dimensions and the EQ VAS] and absenteeism [iMTA Productivity Cost Questionnaire (iPCQ; Bouwmans et al., 2015)]. HCPs were asked to complete the socio-demographical and job characteristics questionnaire and other questionnaires about quality of care, job satisfaction (report mark), job efficiency (report mark) and job demand (report mark).

At one year and two years, the patients and HCPs received the same questionnaires as they received during baseline, except for the socio-demographical and clinical characteristics questionnaire. Furthermore, they both received questionnaires about the usage of the PHR, and their satisfaction with the PHR [Client Satisfaction Questionnaire (CSQ-8; Larsen et al., 1979), System Usability Scale (SUS; Brooke, 1996) and report mark]. Only at the one-year mark, they needed to fill in a questionnaire about the determinants of the PHR [Measurement Instrument for Determinants of Innovations (MIDI; Fleuren et al., 2014)].

Patients and HCPs who were interested in participating in the focus group interview were contacted by email after two years to schedule the focus group via a planning tool (Datumprikker, 2022). An interview protocol was developed based on the Unified Theory of Acceptance and Use of Technology (UTAUT) framework (Venkatesh et al., 2003). The focus group with the patients was organised at the LUMC and lasted two hours, while the focus group with HCPs was organised online via Microsoft Teams and lasted 45 min. They received a gift card of 25 euros and their travel costs were reimbursed when applicable.

### Outcome measures

#### Socio-demographical and clinical characteristics

For patients, the following information was obtained: gender, age, educational level, country of birth, working status or other daytime activities, living situation, type of MS, and years and months since the MS diagnosis. For HCPs, the following information was collected: gender, age and job function within the hospital.

#### Feasibility and usability

Both patients and HCPs filled out questionnaires about usage, usability and satisfaction with the PHR. Usage of the PHR was measured with a self-reported questionnaire about the frequency and duration of usage, as well as the reason for using the PHR. Patients and HCPs had the option to choose several reasons for utilizing the PHR, and they also had the opportunity to respond in an open-ended format if their rationale deviated from the provided options.

Usability was measured with the SUS (Brooke, 1996) consisting of 10 items rated on a five-point Likert scale ranging from strongly disagree (0) to strongly agree (4). The scores were multiplied by 2.5 to obtain the total score ranging from 0 to 100. A higher score meant that the PHR was more user-friendly. Scores of 68 or higher are considered above average, indicating that patients and HCPs find the PHR usable.

The CSQ-8 (Larsen et al., 1979) examined the satisfaction with the PHR. The questionnaire contained eight items with four answer categories ranging from, for example, poor (1) to excellent (2). Four of the eight items were reversed scored, and afterwards, the scores were summed. Total scores ranged from 8 to 32, with higher scores indicating greater satisfaction.

The focus group interviews were conducted to qualitatively assess the feasibility and usability of the PHR and to explore the secondary outcomes (i.e., implementation determinants and preliminary effects), utilizing the UTAUT framework (Venkatesh et al., 2003). The UTAUT framework identifies four primary factors influencing the intention and usage of technology, in this study specifically in the context of a PHR: (1) performance expectancy, (2) effort expectancy, (3) facilitating conditions and (4) social influence (Venkatesh et al., 2003). Performance expectancy refers to the general benefits associated with using the PHR and the feasibility of the PHR. Effort expectancy is the ease of use and usability of the PHR. Facilitating conditions means that there are sufficient resources and knowledge to use the PHR. Social influence is the influence of other people (e.g., family, friends, acquaintances, HCPs) to start and keep using the PHR, and whether they would recommend the PHR to others. Additionally, the UTAUT framework incorporates four moderating factors: (1) gender, (2) age, (3) experience and (4) voluntariness of use (Venkatesh et al., 2003). These factors, along with the moderating factors, were topics of discussion during the focus group interviews.

#### Implementation determinants

The implementation determinants of the PHR were assessed using four items on the subscale ‘Innovation’ of the MIDI (Fleuren et al., 2014) for the patients. The HCPs received five items on the subscale “Innovation,” eight items on the subscale “End-user,” and 10 items on the subscale “Organization.” These items were pre-selected by the research team and consisted of determinants which were not collected via the other outcome questionnaires. Answers were rated on a five-point Likert scale ranging from, for example, totally disagree (1) to totally agree (5). Three determinants, related to the ‘Organisation’ subscale, were discussed with a dichotomous scale. Items answered by ≥20% of the patients or HCPs with “totally disagree/disagree” were considered barriers, and items answered by ≥80% of the patients or HCPs with “totally agree/agree” were considered facilitators (Schepers et al., 2017).

Furthermore, both patients and HCPs received a question assessing their general attitude toward using the PHR in their treatment or work environment, respectively. This was rated on a five-point Likert scale ranging from very negative (1) to very positive (5). Additionally, they were asked to respond to two open-ended questions about the positive and negative effects they experienced or expected in the upcoming period.

#### Preliminary effects

The experienced quality of care was measured with the questionnaire “experienced quality of care” consisting of 15 questions on a 10-point scale ranging from very little (1) to very well (10) with questions such as “How do you review the quality of care you received from the MS-centre of the *Alrijne* Hospital?” The questionnaire is partly based on an earlier patient satisfaction survey performed at the MS-centre of the *Alrijne* Hospital and partly based on the consumer quality index (Delnoij et al., 2010). Mean scores were computed ranging from 1 to 10, with higher scores indicating a better-experienced quality of care (*α* = 0.969).

Additionally, patients received questions about self-management. The PIH (Battersby et al., 2003) consisted of 12 items which measured different domains of self-management: knowledge, coping, monitoring of symptoms, and proactive role during the consultation. Each item was rated on a nine-point scale from very little (0) to a lot (8). An example of an item was “In general, I know a lot about my condition.” A higher score indicated a greater level of experienced self-management.

Self-efficacy was also measured with the “Control” subscale from the MSSE (Schwartz et al., 1996), consisting of six items instead of the original nine items after a validation study (Chiu and Motl, 2015). This subscale assessed the confidence in one’s ability to deal with the disease and the symptoms of MS, making adjustments to improve living with MS, and the impact of the disease on daily activities. The items were answered on a scale from feeling very uncertain (10), to moderately certain (50) and very certain (100). A total score was created by the summation of the items, with higher scores indicating more self-efficacy.

HCPs also received questions about job satisfaction, job efficiency and job demand. These factors were examined with a report mark ranging from 1 to 10. A higher score indicated more satisfaction, efficiency, or demand.

#### Costs and health-related quality of life

The IMCQ (Erasmus University Rotterdam, 2023) was used to measure the healthcare usage of the patients. For the current study, questions were selected about the number of consultations with various HCPs (e.g., general practitioners, neurologists, physical therapists, urologists). In the original questionnaire, the number of consultations in the last three months was asked; however, patients with MS visit the MS-centre sometimes only once a year, as part of usual care. Therefore, we changed the period in all questions which were hospital-related from three months to 12 months. In addition, patients completed the iPCQ (Bouwmans et al., 2015) about absenteeism, presenteeism and decreased productivity resulting from unpaid activities such as household chores or volunteering.

Health-related QoL was assessed with the EQ-5D-5L descriptive system and EQ VAS (EuroQoL Research Foundation, 2019). The EQ-5D-5L consisted of five dimensions (i.e., mobility, self-care, usual activities, pain/discomfort and anxiety/depression) and each dimension could be rated on five levels of severity from no problems (1) to extreme problems (5). A lower score indicated having fewer problems. The EQ VAS consisted of a visual analogue scale about the patient’s subjective health perception of the day they filled out the questionnaire. They could indicate their health on a scale from the worst health they could imagine (0) to the best health they could imagine (100).

The Dutch tariff (Versteegh et al., 2016) was used to convert EQ-5D-5L scores into utilities, i.e., a value between 0 (equal to death) and 1 (perfect health). EQ VAS scores were divided by 100 to obtain utilities. Furthermore, the number of visits to each healthcare provider was multiplied by its cost price as indicated in the Dutch guidelines for costing studies (Hakkaart-van Roijen et al., 2015; Kanters et al., 2017). Absenteeism costs were calculated by multiplying the number of hours missed in the last four weeks, extrapolated to one year, by the average gross hourly wage of working people in the Netherlands (Hakkaart-van Roijen et al., 2015). For longer absences due to sickness, the friction cost method was applied, i.e., no costs were incurred after 12 weeks of absence, as it was expected that the initial level of production would have been restored by then. Presenteeism costs were calculated by multiplying the number of hours of reduced productivity due to health problems in the last four weeks, extrapolated to one year, by the average gross hourly wage of working people in the Netherlands (Hakkaart-van Roijen et al., 2015). Costs related to reduced productivity of unpaid work were calculated by multiplying the recalled hours in which others had to do the work instead of the participant in the last 4 weeks, extrapolated to one year, by the average gross hourly wage of a houseworker (Hakkaart-van Roijen et al., 2015). All costs were indexed to the year 2023 using the Dutch consumer price index (StatLine, 2024).

### Statistical analysis

The quantitative data were analysed using SPSS (version 25.0) (IBM Corp, 2017). For all study aims, descriptive analyses were used, reporting means, standard deviations, *N*, and percentages. Furthermore, linear mixed models were conducted to examine the preliminary effects of the PHR. For each preliminary effect, a separate linear mixed model was run, with the outcome measurement as the dependent variable, time as a fixed effect, and patients as a random effect. The preliminary effects of the HCPs were not examined via linear mixed models because insufficient data were collected.

The two focus group interviews were audio-recorded, which were transcribed intelligent verbatim by researcher LK. Atlas.ti version 22.0 (ATLAS.ti, 2023) was used to code and analyse the data according to the principles of the Framework Method (Gale et al., 2013). This was done by researchers LvdB and LK. Upon completion of the transcription phase, both researchers engaged in the interview transcripts to establish a comprehensive understanding of the content. A deductive approach was used to code the interviews based on a predefined codebook, which was developed in advance, based on the UTAUT framework (Venkatesh et al., 2003). When necessary, additional codes were incorporated into the codebook. The coding process was independently executed by LvdB and LK, followed by a comparison of the codes. A framework matrix was used to effectively manage, analyse and summarise the data, highlighting relevant quotes from both patients and HCPs. Finally, the researchers identified distinctive characteristics and patterns within the dataset. The codebook, codes and data analysis were both discussed with researcher JA, who supervised the process.

## Results

### Socio-demographical and clinical characteristics

#### Patients

Seventy-five of the 80 included patients completed the baseline questionnaire. An overview of the socio-demographic and clinical characteristics can be found in Table 1. The majority of the patients were female (68.0%) and had a mean age of 53 years (*SD* = 10.3). Most patients were born in the Netherlands (89.3%), had a secondary vocational education or higher (73.4%), had a paid job (50.6%), and lived together with a partner or family (81.4%). Furthermore, most patients had relapsing–remitting MS (53.3%). On average, the number of years since the MS diagnosis was 13.5 (*SD* = 11.6).

**Table 1:** Baseline socio-demographical and clinical characteristics of the patients in the quantitative measurements.

Characteristic	Value (*n* = 75)
*Gender [n (%)]*	
Male	24 (32.0)
Female	51 (68.0)
Age [M (SD) [range]]	52.8 (10.3) [28–75]
*Educational level [n (%)]*	
Secondary education	20 (26.7)
Secondary vocational education	20 (26.7)
Higher professional education	24 (32.0)
University education	11 (14.7)
*Country of birth [n (%)]*	
The Netherlands	67 (89.3)
Other (i.e., Germany, France, Iran, Italy, Lithuania, Serbia)	8 (10.7)
*Working status or other daytime activities [n (%)]*	
Fulltime paid job	16 (21.3)
Parttime paid job	22 (29.3)
Volunteer work	3 (4.0)
Retired	7 (9.3)
Incapacitated	21 (28.0)
Sickness benefit	1 (1.3)
Other (i.e., no job, housekeeping, other benefit, taking care of a child, self-employed)	5 (6.7)
*Living situation [n (%)]*	
Living together with a partner	32 (42.7)
Living together with a partner and child(ren)	29 (38.7)
Student housing or living with friends	2 (2.7)
Alone	9 (12.0)
Other (i.e., alone with children, partly living together)	3 (4.0)
*Type of MS [n (%)]*	
Relapsing–remitting MS (RRMS)	40 (53.3)
Secondary progressive MS (SPMS)	6 (8.0)
Primary progressive MS (PPMS)	11 (14.7)
Undetermined	2 (2.6)
I do not know	16 (21.3)
Years since MS diagnosis [M (SD) [range]]	13.5 (11.6) [0.3–43]

M, mean; SD, standard deviation; n, sample size; MS, multiple sclerosis; RRMS, relapsing–remitting MS; SPMS, secondary progressive MS; PPMS, primary progressive MS.

In the focus group interview, four women (57.1%) and three men (42.9%) participated with a mean age of 59 (*SD* = 8.2). The number of years since the MS diagnosis ranged between 3 and 55 years.

#### HCPs

Twelve HCPs completed the baseline questionnaire. The majority was female (83.3%) and the mean age was 49 years (*SD* = 10.3). Most HCPs worked as a neurologist (41.7%), followed by having a job as an MS nurse (17%) or a physiotherapist (17%).

In the focus group interview, three women and one man participated. Two HCPs had a job as a neurologist, one as a physician assistant and one as a urologist. The number of years they worked at the participating hospital ranged between 7 and 27 years.

#### Feasibility and usability

##### Patients

**Figure 1:**
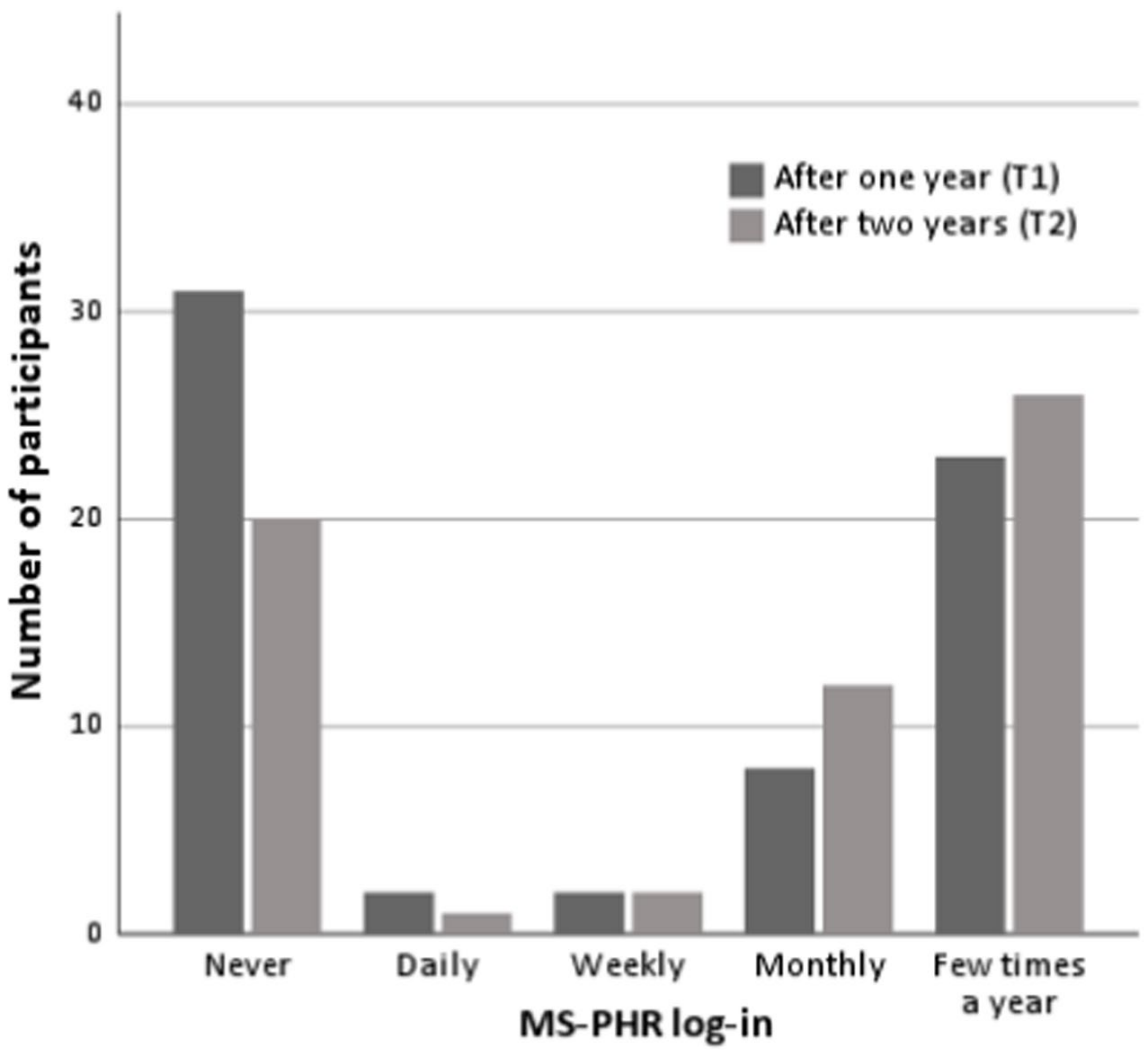
Number of self-reported log-ins.

Figure 1 shows an overview of the self-reported log-in numbers. At T1, most patients reported to have never logged in (31/66, 47.0%). At T2, the majority stated that they logged in a couple of times per year (26/61, 42.6%). On average, patients used the PHR for 15 min (*SD* = 11.2, range 2–45) per log-in moment after 1 year, and for 17.7 min (*SD* = 13.1, range 2–60) after 2 years.

They self-reported to mainly use the PHR to view data provided by their HCP (T1: 18/35, 51.4%; T2: 18/41, 43.9%) and to fill in the EQ-5D questionnaire (T1: 18/35, 51.4%; T2: 25/41, 61.0%). Furthermore, patients reported using the PHR because they wanted to fill out the rest diary (T1: 6/35, 17.1%; T2: 7/41, 17.1%), medication usage (T1: 4/35, 11.4%; T2: 6/41, 14.6%), and to use the digital decision aid “Do I have a relapse?” (T1: 1/35, 2.9%; T2: 2/41, 4.9%). Other reasons (T1: 11/35, 31.4%; T2: 6/41, 14.6%) were to check medical test results, communicate with HCPs, look up appointments, and keep track of the condition, symptoms and disease process over time.

On average, patients rated their overall experience with the PHR with a report mark of 6.6 (*SD* = 1.5, *n* = 33) at T1, and 6.8 (*SD* = 1.6, *n* = 37) at T2. Usability was below average on both time points, with SUS-scores of 59.9 (*SD* = 14.2, *n* = 33) after one year, and 59.0 (*SD* = 16.3, *n* = 37) after two years. Satisfaction with the PHR was moderate at both time points, with CSQ-8 scores of 21.4 (*SD* = 5.0, *n* = 34) at T1, and 22.1 (*SD* = 5.0, *n* = 39) at T2.

Qualitative data (*n* = 7) indicated that none of the patients had prior experience with a PHR. Four patients (57.1%) mentioned that they had used their electronic patient portal (i.e., *mijnAlrijne*) before using the PHR. Two patients expressed satisfaction with the development of the PHR, especially due to ongoing modifications which resulted in enhanced clarity of the PHR. Initially, both patients found the questions in the questionnaire occasionally unclear and ambiguous, and the PHR lacked a structured format.

One patient used the PHR twice per month and another patient three times per month for circa 15 min per log-in moment, while four others logged in between one to six times per year. Another patient had not used the PHR at all.

Regarding “performance expectancy,” the majority of the patients expressed the belief that the PHR could contribute to enhancing a multidisciplinary care approach for individuals with MS. They also appreciated the capability of HCPs to access the health data entered into the PHR. Additionally, they thought that the PHR has the potential to show information and trends in their health and health behaviors over time.

“I have sleeping problems for instance, but I go late to bed as well. So I am responsible. When the PHR can show me this information, there is an added value.” – Female patient, 57 years.

There were also negative aspects pointed out by the patients. For instance, they mentioned that some questionnaires should be tailored, as the pregnancy questionnaire was displayed to everyone instead of only being presented to women within a specific age range.

“I am bothered by the fact that there are questionnaires that I must fill out, that every time I have to fill out the questionnaire on pregnancy as well.” – Male patient, 68 years.

In terms of “effort expectancy,” one patient stated that the information in the PHR was not always up-to-date. For instance, his medication was changed, but this was not visible in the PHR and was experienced as annoying. Furthermore, four patients mentioned a graphical overview per question would help them to compare improvements and setbacks over time. This way, the course of the disease can be taken into account and insights can be provided on long-term well-being. Currently, two patients mentioned that they occasionally questioned the responses they provided in the last questionnaire, as the answer scales could be interpreted in various ways. Moreover, the responses to the questionnaire are contingent on the moment and can easily vary from day to day.

“Filling out the questionnaire is like taking a snapshot, and sometimes I find myself wondering: how did I interpret this the last time I filled it out?” – Female patient, 49 years.

Regarding “facilitating conditions,” it would be helpful for the patients to easily provide feedback if something within the PHR is not working. This was a missed functionality because they mentioned that they already had tried to provide feedback about, for example, the pregnancy questionnaire (i.e., being displayed to everyone), but this was not changed yet in the PHR. Exchanging their experiences with the PHR helped them to become more motivated and enthusiastic about the PHR.

“It was great to speak with other patients with the same problems and what the PHR can contribute. It piqued my interest.” – Female patient, 57 years.

Regarding “social influence,” the six patients who use the PHR initiated its use upon recommendation of their neurologist, or out of motivation to provide health information data to their HCPs. The patient who did not use the PHR mentioned that her neurologist did not recommend it to her.

“The neurologist does not use the PHR because she does not see the value for me and does not see what can be in it for me.” – Female patient, 57 years.

##### HCPs

After one year, most HCPs logged in a couple of times per year (*n* = 4), followed by never logged in (*n* = 3), and logged in weekly (*n* = 2). After two years the distribution was: logged in a couple of times per year (*n* = 2), never logged in (*n* = 2), and logged in weekly (*n* = 1) or monthly (*n* = 1), respectively. On average, HCPs used the PHR for 6 min (*SD* = 2.2, range 5–10) per log-in moment after 1 year, and for 6.8 min (*SD* = 5.7, range 2–15) after 2 years.

They generally used the PHR to view the provided data from the electronic patient portal (T1: 3/6; T2: 2/4) and to view patients’ completed questionnaires (e.g., EQ-5D) before a consultation with the patient (T1: 3/6; T2: 1/4). Other reasons (T1: 1/6; T2: 1/4) were to register patients to the PHR, to set up the communication system within the PHR, and to enter patients’ measurement data in the PHR.

HCPs assigned their overall experience with the PHR a report mark of 6.3 (*SD* = 0.6, *n* = 3) after 1 year of usage, and 6.7 (*SD* = 0.6, *n* = 3) after 2 years. Usability was below average on both time points, with SUS-scores of 53.3 (*SD* = 12.6, *n* = 3) at T1 and 62.5 (*SD* = 10.9, *n* = 3) at T2. CSQ-8 scores demonstrated moderate satisfaction with the PHR (T1: *M* = 20.7, *SD* = 2.5, *n* = 3, and T2: *M* = 22.0, *SD* = 3.2, *n* = 4).

During the focus group interview, three out of four HCPs mentioned that the concept of the PHR is great and relevant for the future. However, all HCPs no longer used the PHR or only had opened the PHR once or a couple of times (see “preliminary effects”).

Regarding “performance expectancy,” the PHR does not meet the HCPs’ expectations yet. Although three HCPs mentioned that the PHR could be useful for patients to get more insight into their disease progression and gain more autonomy.

In terms of “effort expectancy,” the HCPs expressed that the graphs within the PHR are not always clear, and the information is not always easily accessible, both crucial aspects for efficient utilization of the PHR. Furthermore, one HCP explained that an extra activity has to be done because the patient provides information via a new system, which is not yet linked with the hospital information system, and this takes too much time. A more compact PHR with minimum information could provide this, with less noise, as it is difficult to find the needed information within the PHR when working with the PHR.

“Include a few buttons or pop-up options in the PHR that show you in ten seconds: ‘I have to do something with this.” – Female HCP.

Regarding “facilitating conditions,” the focus group interview was perceived as a valuable resource. Two HCPs mentioned that it offered a way to provide feedback on the PHR, thereby emphasizing the significance of having a dedicated point of contact.

“I think it is great to have this meeting since you can hear what we expect from the PHR.” – Female HCP.

The “social influence” was not discussed during the focus group interview by the HCPs.

### Implementation determinants

#### Patients

The biggest group of patients were neutral or slightly positive (44/60, 73.3%) about the PHR (see Table 2). The positive consequences they experienced or anticipated were gaining more insight into their disease progression, enhanced insight into their health data and health data from the HCPs / gaining a useful overview, improved self-management, convenient and secure contact with HCPs, and improved information provision.

**Table 2:** Results of patients of the question: “In general, how do you feel about using a PHR in the care/treatment you receive?”

Option	Value [*n* (%)]
Very positive (I am enthusiastic/I am/want (to) using/use the PHR)	8 (13.3)
Positive (slightly enthusiastic)	21 (35.0)
Neutral	23 (38.3)
Negative (I am reserved/hesitant)	1 (1.7)
Very negative (I do not see the point / I do not want to use the PHR)	7 (11.7)

The negative consequences they experienced or anticipated were technical difficulties (e.g., log-in problems, problems with the questionnaires or structure of the PHR), receiving irrelevant questionnaires or too many questionnaires, facing unnecessary confrontation with their disease progression, too much focus on the disease instead of recovery or prevention (i.e., promoting healthier lifestyle), and reduced face-to-face interactions with others.

**Figure 2:**
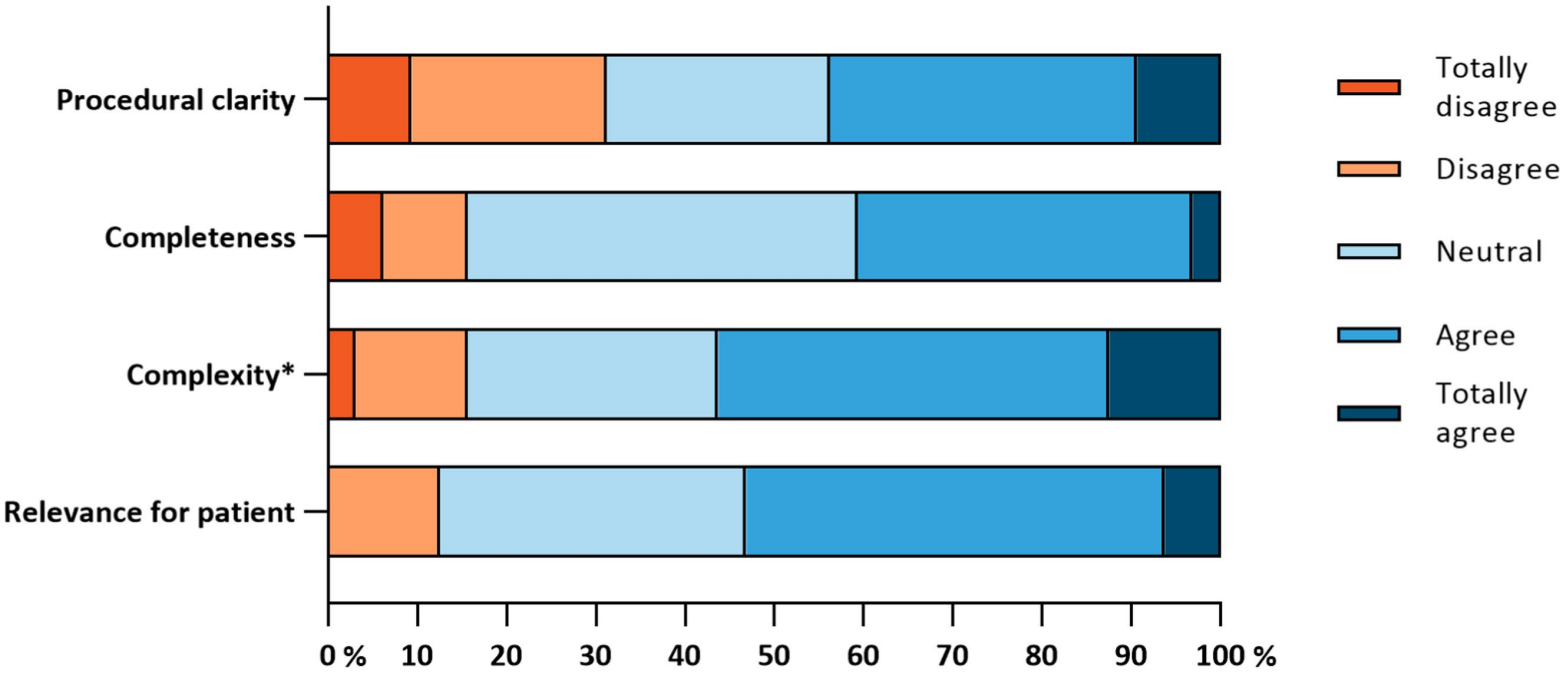
Implementation determinants of the “Innovation” subscale (*n* = 32). *This item was reversed, the original item was: “The PHR is too complex for me to use”.

Only one barrier was identified (10/32, 31.3%), according to the MIDI cut-off points (≥20%), namely the procedural clarity of the PHR (see Figure 2). It was unclear to patients in which order they needed to perform which activities in the PHR. No facilitators were identified by the patients.

Multiple barriers were identified by the patients in the focus group interview, especially in terms of “effort expectancy.” One issue was the unclear language used within the PHR. Additionally, patients encountered confusion about how to use the PHR in general, and the goal of using the PHR was not always clear. Moreover, four patients faced log-in issues, as they could not easily locate the log-in link in the invite email. The use of multiple names for the PHR contributed to the difficulty in remembering which name was required to find the link. Patients suggested that improved explanations (i.e., “facilitating conditions”) about the log-in procedure and guidance on saving the necessary link could address these log-in issues.

“To use the PHR you received a link from the Alrijne Hospital. Without the link, it is absolutely not possible to log-in.” – Male patient, 68 years.

Patients also discussed a possible facilitator to start using the PHR, regarding “performance expectancy,” namely the fact that the PHR can provide more insight into health information. Moreover, they mentioned several recommendations regarding the implementation process: more features should be added that are useful for the patients, this will motivate them to use the PHR regularly and could provide more insight into one’s health data. Six patients noted that using the PHR on a smartphone was challenging, as it was initially designed for computer use. Patients suggested that having a dedicated PHR app would be beneficial. Additionally, eliminating the need for a log-in link would further assist the patients. Ultimately, a clearer explanation of the PHR, including its functionality, purpose, user eligibility, and visibility of health information, would be beneficial for them. This information should be presented in a clear and straightforward manner.

“When more features are added in the PHR and more is possible with the PHR that could be of value for me as well, then I would like to get an explanation on this.” – Female patient, 49 years.

#### HCPs

Four of the six HCPs (66.7%) had a positive or very positive attitude to the PHR, whereas the remaining two (33.3%) had a negative attitude. The positive consequences they experienced or anticipated were gaining a better overview (i.e., of the disease progression, medication usage, tests of the patients, impact of the MS on the daily life) and accessing information about the health of the patient prior to a consultation.

The negative consequences they experienced or anticipated were the time burden of working with the PHR for both themselves and the patients (e.g., more administration, too many questionnaires) and technical difficulties.

**Figure 3:**
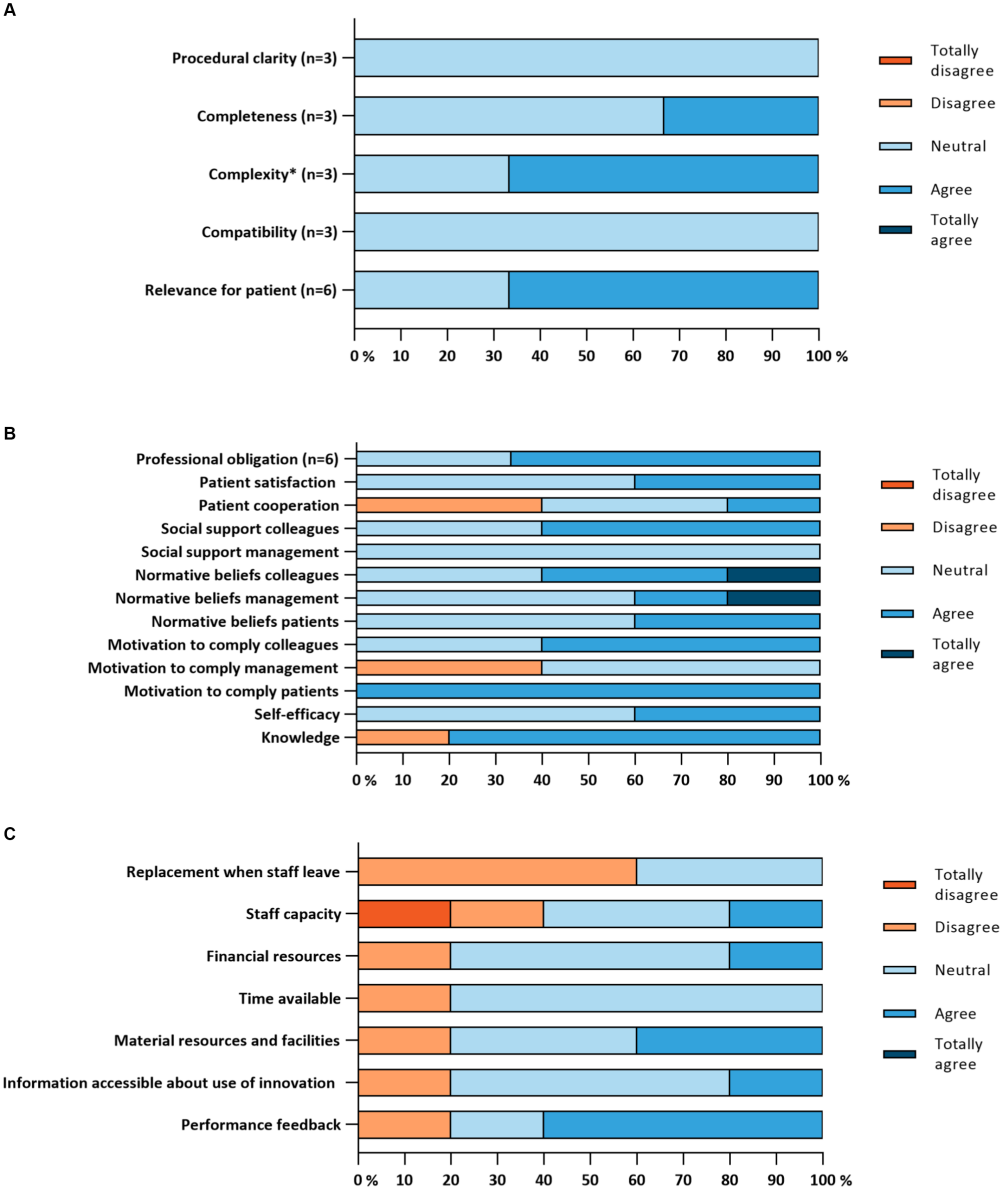
**(A)** Implementation determinants of the “Innovation” subscale. **(B)** Implementation determinants of the “End-user” subscale (*n* = 5). **(C)** Implementation determinants of the “Organization” subscale (*n* = 5). ^*^This item was reversed, the original item was: “The PHR is too complex for me to use”.

None of the determinants from the ‘Innovation’ subscale were seen as barriers or facilitators by the HCPs, according to the MIDI cut-off points (≥20% for barriers and ≥ 80% for facilitators) (see Figure 3A). Most of them were neutral about the statements.

HCPs reported that a minority of their colleagues for whom the PHR was intended, actually was using the innovation. The results concerning the other determinants are shown in Figure 3B. Three barriers were identified by the HCPs, according to the MIDI cut-off point (≥20%), namely concerns about patients not being willing to cooperate to use the PHR (2/5, 40.0%), when it comes to working with the PHR, the opinion of the management does not matter much to them (2/5, 40.0%) and the lack of sufficient knowledge to use the PHR (1/5, 20.0%). This last barrier was also identified as a facilitator when sufficient knowledge is experienced (4/5, 80%). Another facilitator was the fact that the opinion of patients does matter to them when it comes to working with the PHR (5/5, 100.0%).

All organizational determinants were seen as barriers, according to the MIDI cut-off points (≥20%), during the implementation process of the PHR (see Figure 3C). One dichotomous item showed the absence of formal protocols in the hospital relating to the usage of the PHR, which can also be seen as a barrier. However, two dichotomous items showed potential facilitators, namely: a designated person or multiple persons is/are responsible for coordinating the implementation process, and there are no other changes in the hospital which could affect the implementation or the usage of the PHR at the moment or in the foreseeable future.

One HCP, in the focus group interview, no longer used the PHR anymore, because it became demotivating when a large number of patients did not provide any input in the PHR. Other barriers, primarily related to “effort expectancy,” were also identified and discussed during the focus group interview: the separate nature of the PHR program, the abundance of information within the PHR, difficulty in retrieving useful information due to the lack of pop-up functionality, and issues with the PHR not working as intended, resulting in HCPs being put on hold for its use.

“The PHR should be completely ready to use before it is going to be implemented.” – Female HCP.

“I will not look in the system to know when there was a relapse. I look that up in my information. […] When you quickly see which information is relevant in the PHR, I am willing to open it to see whether something is alarming in the PHR.” – Female HCP.

No facilitators were identified during the focus group interview. It was essential for the HCPs that the PHR should be adjusted to practice and that it should be more easy to use.

“The PHR should be adjusted to practice so that you can see the most relevant patient information easily before the consultation.” – Male HCP.

### Preliminary effects

#### Patients

The experienced quality of care of patients was good at all three-time points, with scores of 8.4 (*SD* = 1.0, *n* = 75) at T0, 8.2 (*SD* = 1.5, *n* = 56) at T1, and 8.2 (*SD* = 1.2, *n* = 54) at T2. The linear mixed model analysis showed no significant effect over time of the PHR on the experienced quality of care (*β* = −0.08, *SE* = 0.07, *p* = 0.259).

Regarding self-management, patients scored on average 82.4 (*SD* = 7.8, *n* = 75) on the PIH at baseline, and 83.0 (*SD* = 7.8, *n* = 54) and 82.8 (*SD* = 8.4, *n* = 50) after one year and two years, respectively. No significant effect over time of the PHR on self-management was found (*β* = 0.49, *SE* = 0.54, *p* = 0.369).

Self-efficacy, measured with the MSSE, was above average at all three-time points, with scores of 464.9 (*SD* = 89.7, *n* = 75) at T0, 468.1 (*SD* = 109.6, *n* = 54) at T1, and 467.0 (*SD* = 95.9, *n* = 50) at T2. The linear mixed model showed no significant difference over time for self-efficacy (*β* = 2.23, *SE* = 5.32, *p* = 0.675).

In terms of “performance expectancy,” the patients expressed that the PHR had the potential to enhance the quality of care by providing additional information to the neurologist. For example, the HCP could access the patient’s health data prior to consultations. Additionally, a patient noticed a shift in his relationship with the neurologist and MS nurse, as many inquiries no longer needed to be posed since the relevant information was already available in the PHR.

“Of course, because otherwise, she would have to ask a hundred questions, but now she has already seen them answered over time.” – Male patient, 68 years.

“You provide information before the consultation. This way, the HCP can already read the information.” – Male patient, 49 years.

Moreover, four patients expressed that they think a PHR could help with applying a multidisciplinary approach in the healthcare of MS patients. This could be further improved by giving other HCPs from other healthcare institutions access to the PHR to view the patient’s health information. Moreover, a list with all HCPs should be added in the PHR so that patients can easily select who has access to their health data. The added value of the PHR is directed towards the HCPs and not necessarily to the patients themselves.

“For me, that is the motivation to fill out the PHR. To provide the HCP with information to see ‘that is how I stand’. What can be improved? Or what can be changed? How can he be helped?” – Male patient, 49 years.

One patient also stated that the PHR could improve self-management by providing greater insight into the experienced symptoms. The content within the PHR might guide patients in the right direction to seek help.

Regarding “effort expectancy,” two patients mentioned that they do not always want to fill in how they feel at the moment and that is not always a priority to fill in the questionnaires in the PHR.

#### HCPs

HCPs experienced the quality of care at the hospital they worked at as good at all three-time points: 7.9 (*SD* = 0.6, *n* = 12) at T0, 8.8 (*SD* = 0.5, *n* = 5) at T1, and 8.4 (*SD* = 0.4, *n* = 5) at T2.

Regarding job satisfaction, at baseline HCPs scored on average a 7.6 (*SD* = 0.5, *n* = 12), a 8.2 (*SD* = 0.4, *n* = 5) after one year and a 7.6 (*SD* = 0.5, *n* = 5) after two years. Similar results were obtained in terms of job efficiency scores: 7.6 (*SD* = 0.7, *n* = 11) at baseline, 8.0 (*SD* = 0.7, *n* = 5) after one year and 7.6 (*SD* = 0.5, *n* = 5) after two years. The job demand was perceived as relatively moderate throughout the study period: 7.1 (*SD* = 1.3, *n* = 12) at baseline, 7.8 (*SD* = 0.8, *n* = 5) after one year, and 6.8 (*SD* = 0.4, *n* = 5) after two years.

Regarding “performance expectancy,” the HCPs expressed that the PHR might be helpful as a communication system between HCPs and patients. It could also provide HCPs with valuable information about the disease progression of the patients, and patients could be more informed and get more insight into their disease progression themselves. One HCP mentioned that prospectively collecting health data through the PHR could help to gain insight into the whole patient population related to the worsening of the disease. Moreover, the PHR could help to indicate when something is notably different in terms of disease progression. Furthermore, under specific circumstances, for example when communication is not clear or difficult, it is possible that information is missed by a HCP that could successfully be captured and provided in questionnaires in a PHR.

“When you can tell a large group of patients ‘Pay attention, when you have COVID-19 you have to call us to get specific medicines.’ You can reach out to a lot of patients via the PHR.” – Female HCP.

“I can imagine that under certain circumstances, especially when the communication is not clear enough, that a HCP misses information which can be given via the PHR questionnaires.” – Male HCP.

Furthermore, HCPs mentioned that it is crucial to incorporate expectation management regarding the PHR usage to avoid placing excessive pressure on HCPs.

“When a patient fills out the PHR and we do not do anything with it, the quality of care decreases.” – Female HCP.

During the focus group interview, two HCPs mentioned that the PHR could contribute to higher job efficiency. Furthermore, it could also save time during a consultation, because of the usage of standardized questionnaires.

### Costs and health-related quality of life

Utility scores, which represent patients’ QoL, appeared to decrease slightly over the 2-year follow-up period (see Supplementary Figure 1). The EQ VAS scores, which represent the patients’ own overall assessment of their health, were lower than the EQ-5D utility scores, which are restricted to patient-reported outcomes in five dimensions and are valued by the general public.

A small, non-significant decrease in healthcare costs is observed during the follow-up period (see Supplementary Figure 1). Healthcare costs consist mainly of physiotherapy costs. No clear pattern is observed for productivity costs, which consist of costs of absence from work, costs of being present at work but with reduced productivity (presenteeism costs) and costs of reduced productivity in unpaid work.

The patients mentioned during the focus group that the PHR would not influence their QoL, or their work or unpaid work. It was difficult for them to say what the effect of the PHR would be on healthcare consumption since MS is unpredictable.

“I think that the question regarding healthcare consumption is not relevant for MS. With MS you get a relapse and that is a low point and then you go to your specialists. You do not know this beforehand and the PHR cannot do anything about this.” – Male patient, 49 years.

Three HCPs stated during the focus group that the PHR might affect QoL. One HCP mentioned that the PHR can only affect absenteeism or whether patients can do voluntary work when trends become visible to patients in the graphical overview. This may result in a more positive perspective.

“When patients pay attention to things that were unknown at first, then it may result in an improved quality of life.” – Male HCP.

Only the urologist among the HCPs thought that the PHR could contribute to fewer consultations per year.

## Discussion

### Principal results

PHRs provide a digital aid to support patients with their disease by gathering disease and health-related data. The expectation is that, by exchanging data from the PHR and the hospital information system, patients and HCPs are better informed and have more information via an overview of the disease course. Besides, self-management and self-efficacy might be enforced. Unnecessary hospital visits might be avoided and the quality of care might be improved. The present study aimed to assess the feasibility and usability of a PHR for patients with MS implemented in a Dutch hospital. Additionally, the implementation determinants were evaluated and preliminary effects on quality of care for both patients and HCPs, self-management, self-efficacy for patients, job satisfaction, efficiency, and demand for HCPs, and preliminary effects on costs and health-related QoL were explored. Overall, feasibility and usability scores were below average with moderate usability and satisfaction scores, indicating persistent challenges for both patients and HCPs who used the PHR. We chose to provide patients only with a paper with user instructions. More guidance might have improved these results. Both groups acknowledged the benefit of gaining insights into disease progression, but faced technical difficulties that diminished the system’s user-friendliness. Notably, no significant differences over time were observed in patients regarding the preliminary effects, and the sample size of HCPs was insufficient to draw meaningful conclusions. Moreover, minimal non-significant changes in QoL and costs over time were observed. However, no control group was included in this study and therefore we do not know whether QoL would have been even lower and costs would have been higher without the PHR.

Previous research has identified that technical difficulties and unclarities regarding PHR use can affect the adoption of a PHR, emphasizing the crucial role of usability (i.e., it should be as easy as possible to use the system) in implementing innovations (Archer et al., 2011; Kruse et al., 2015; Hsieh et al., 2016; Ross et al., 2016). Other studies, examining the implementation of eHealth interventions, have highlighted similar barriers and facilitators (van Gemert-Pijnen et al., 2011; Ross et al., 2016; Tossaint-Schoenmakers et al., 2021; Khalid et al., 2023). More specifically, barriers such as integration challenges of nonroutine processes, time and attention requirements for usage, minimal engagement from HCPs, limited prioritization of the intervention compared to existing initiatives, as well as facilitators, such as engagement and enthusiasm of users, sufficient knowledge about the intervention, the opportunity to provide feedback, and proper guidance concerning usage. Indeed, success in the implementation process is more likely when the intervention aligns with and becomes an integral part of existing organizational goals and workflows (Khalid et al., 2023). Moreover, it is essential to be aware of the complexity of an implementation process (van Gemert-Pijnen et al., 2011; Ross et al., 2016; Tossaint-Schoenmakers et al., 2021; Khalid et al., 2023), and it is necessary to explore the use of context-specific strategies that align with the implementation process phase (Versluis et al., 2020). This study concludes, in line with previous research, that proper guidance is warranted while implementing a PHR. Besides, the goal of the tool should be clear, and the usage and log-in process of the tool should be as easy as possible. Furthermore, HCPs should be involved while exploring the added value of such a tool.

In this study, we observed moderate adoption rates which were also found in other studies (Archer et al., 2011; Logue and Effken, 2012; Kruse et al., 2015). The adoption rates in our study may account for the absence of differences over time in the preliminary effects on quality of care, self-management and self-efficacy. The implementation process exceeded anticipated timelines, with additional complications arising due to the impact of the COVID-19 pandemic, which may have contributed to a lack of differences in the preliminary effects. Furthermore, there were several hick-ups in data exchange during the study which might have frustrated patients and HCPs. Another explanation is the relatively high scores observed at baseline in this study for quality of care, self-management, and self-efficacy. These elevated scores indicate that there was limited scope for improvement in these outcomes (Sorondo et al., 2016).

Utilizing the UTAUT framework (Venkatesh et al., 2003), performance expectancy was positively related to the intention to use and actual usage of the PHR. Most patients expressed their belief that the PHR could contribute to enhancing a multidisciplinary care approach for individuals with MS, enhance the quality of care, and offer more insight into their health and health data. HCPs stated that the PHR might be helpful as a communication system, but it did not meet their expectations yet. Effort expectancy was negatively associated with the intention to use and actual usage of the PHR. This was mainly due to log-in problems, unnecessary or irrelevant questionnaires, complex language use, and experienced difficulties in retrieving useful information when needed. Regarding facilitating conditions, it would be helpful for the patients to have proper guidance for the log-in procedures. Furthermore, both patients and HCPs mentioned that it is useful to have a dedicated point of contact to provide feedback about the PHR. The social influence also played a crucial role in the intention and usage: all patients who used the PHR started using it upon recommendation of their neurologist or out of motivation to provide health information data to their HCPs. The patient who did not use the PHR also stated that her neurologist did not recommend it. Thus, the usage was mainly externally motivated, while previous research has shown that autonomous motivation (i.e., internal motivation) is necessary to increase engagement and maintenance for behavioral change (e.g., getting started and maintaining usage of the PHR) (Ryan et al., 2008).

### Strengths and limitations

This study possessed several strengths and limitations. A significant strength lay in its longitudinal mixed-method design, with qualitative focus group interviews offering a deeper understanding of feasibility, usability, and various implementation determinants. Furthermore, the study participants were just informed by an instruction letter (without personal help), in accordance with daily practice where a lack of time of HCPs prevents dedicated guidance in the usage of such a new tool. The observational setting therefore minimized interference with daily practice dynamics, allowing for comprehensive insights from both patients and HCPs. Additionally, the study demonstrated a low drop-out rate, ensuring a more complete dataset and enhancing the overall data quality.

A limitation of the study was the small sample size, particularly among HCPs. Careful interpretation of the study data is therefore necessary due to limited generalizability of the findings. The small sample size among HCPs was partly because the neurologists involved with the set-up of the PHR within the hospital were not participating in this study, which reduced the HCP recruitment pool. The extended duration of the implementation process, partly due to the COVID-19 pandemic, presented another challenge. Due to this delay, the PHR was not fully adopted yet by patients and HCPs, which may have negatively impacted the preliminary effects of the PHR on different outcomes. Lastly, the study worked with a minimal viable product and a relatively new concept, influencing the feasibility and usability of the PHR, because, for example, not all features functioned properly and HCPs were recommended to temporarily refrain from using the system due to technical difficulties.

### Implications for future research and practice

The PHR that was examined in this study holds potential for enhancement in the future, because almost half of the patients and two-thirds of the HCPs were (very) positive about wanting to use or using the PHR, warranting further evaluation of its feasibility and usability. Engaging relevant stakeholders, such as patients and HCPs in the continuous developing process, via participatory design (Clemensen et al., 2007), could improve both outcomes. For example, it is essential for patients to have clarity about usage, clearer language and a feedback option within the system and for HCPs to easily retrieve information within the system and receive notifications when relevant information is added by the patient in the PHR.

Subsequent longitudinal research could delve into an enhanced iteration, concentrating on optimal implementation strategies for the PHR. A designated implementation team could improve the success of this process (Fixsen et al., 2007). The emphasis should be on identifying pivotal features essential for the PHR and determining its impact on the preliminary effects of this study. Such an approach would facilitate the examination of psychological effects resulting from PHR usage. Additionally, there is a need for a more profound understanding of the factors motivating patients and HCPs to persist in using the PHR and strategies to enhance its added value for both patients with MS and HCPs.

Moreover, it is imperative to factor in costs for the continued implementation of the PHR, as an eHealth innovation is a dynamic product requiring regular evaluation and adjustments as needed. In order to facilitate this, adequate funding is required, as both implementation, development and maintenance involve ongoing costs. A lack of consistent funding poses a barrier to the implementation process (Ahmed et al., 2019; Versluis et al., 2020).

## Conclusion

A personal health record (PHR) for patients with MS was evaluated within the care setting of a Dutch hospital. The usability and feasibility of the PHR were considered moderate by patients and HCPs. Both groups considered the value of the PHR in terms of performance expectancy, by gaining more insight into the disease progression and facilitating communication; however, barriers related to effort expectancy emerged, including log-in issues, challenges with information retrieval, and unclear language use. These obstacles impeded the adoption and uptake of the PHR, but potential solutions lie in continuous development with a participatory design approach and the establishment of a dedicated implementation team.

## Data availability statement

The datasets presented in this article are not readily available because participants did not give consent to share data with other parties. Requests to access the datasets should be directed to LB, l.n.van_den_berg@lumc.nl.

## Ethics statement

The studies involving humans were approved by Medical Ethics Committee of Leiden University Medical Center. The studies were conducted in accordance with the local legislation and institutional requirements. The participants provided their written informed consent to participate in this study.

## Author contributions

LB: Conceptualization, Data curation, Formal analysis, Investigation, Methodology, Resources, Validation, Visualization, Writing – original draft, Writing – review & editing. JA: Conceptualization, Data curation, Funding acquisition, Investigation, Methodology, Project administration, Resources, Supervision, Validation, Writing – review & editing. LK: Formal analysis, Validation, Visualization, Writing – review & editing. RB: Writing – review & editing. MA: Conceptualization, Formal analysis, Methodology, Writing – review & editing. NC: Conceptualization, Funding acquisition, Methodology, Supervision, Writing – review & editing. EH: Conceptualization, Funding acquisition, Methodology, Supervision, Writing – review & editing.
