# Supplementary material for: The feasibility and usability of a personal health record for patients with multiple sclerosis: a 2-year evaluation study
Source: Front Hum Neurosci. 2024 May 22;18:1379780. doi: 10.3389/fnhum.2024.1379780 (PMC11150701; doi:10.3389/fnhum.2024.1379780)
Supplement: Supplementary file 1 [file Data_Sheet_1.PDF]

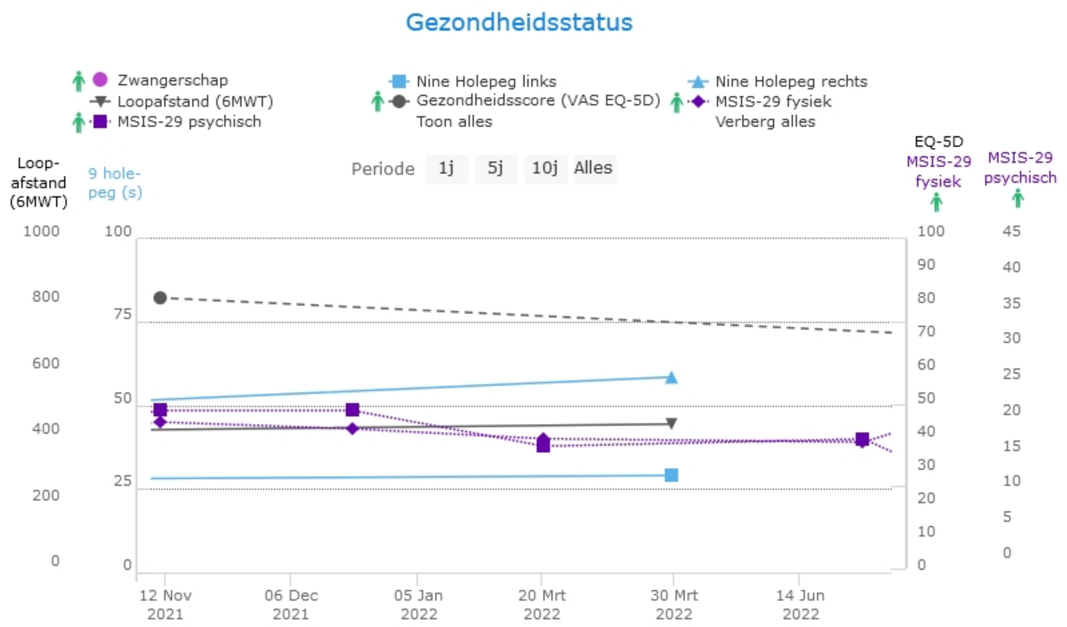


**Figure S1.** Visual of the health status (in Dutch 'gezondheidsstatus') with an overview of clinimetry test results and questionnaire data, including pregnancy status (in Dutch 'zwangerschap'), walking distance (in Dutch 'loopafstand', six minute walk test), the Multiple Sclerosis Impact Scale (MSIS-29, including both the psychological (in Dutch 'psychisch') and physical (in Dutch 'fysiek') score), and the overall health score (in Dutch 'gezondheidsscore') via the VAS EQ-5D.

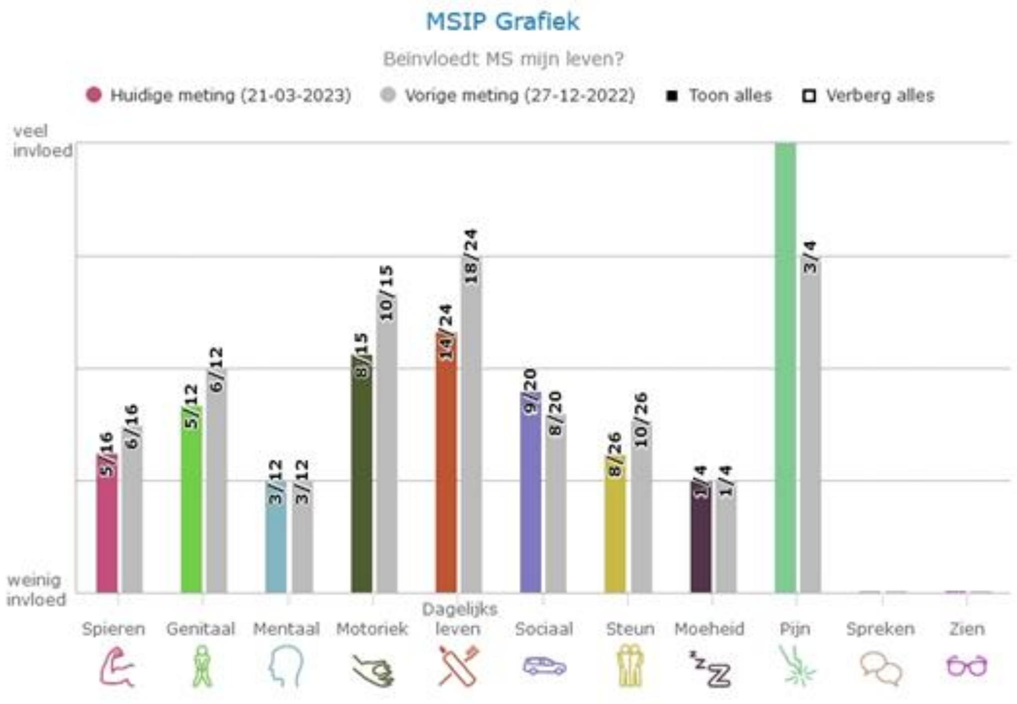


**Figure S2.** Visual of the Multiple Sclerosis Impact Profile (MSIP) graph (in Dutch 'grafiek'), with the question 'does MS influence my life?' (in Dutch 'Beïnvloedt MS mijn leven?'). The

colored bars are the most recent measurement compared to the gray bars which are the results of the previous measurement. This way, patients and HCPs can compare both measurements. Different categories are shown, namely: muscles (in Dutch ‘spieren’), genital (in Dutch ‘genitaal’), psychological (in Dutch ‘mentaal’), mobility (in Dutch ‘motoriek’), daily life (in Dutch ‘dagelijks leven’), social (in Dutch ‘sociaal’), support (in Dutch ‘steun’), fatigue (in Dutch ‘moeheid’), pain (in Dutch ‘pijn’), speaking (in Dutch ‘spreken’), and seeing (in Dutch ‘zien’). The scale goes from little influence (in Dutch ‘weinig invloed’) to a lot of influence (in Dutch ‘veel invloed’).

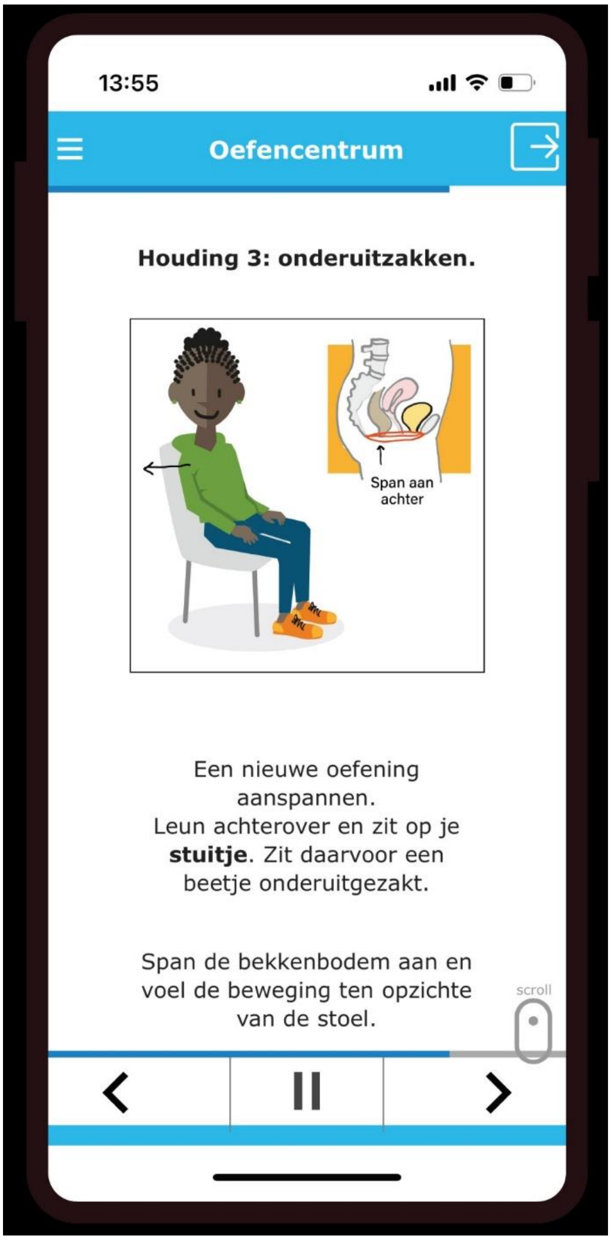


**Figure S3.** The “Uricontrol-module”. The visual shows the practice center (in Dutch ‘oefencentrum’) with a new position (in Dutch ‘houding’) individuals can practice. In this case, individuals try out a new exercise “tightening” (in Dutch ‘een nieuwe oefening aanspannen’). They should lean back and sit on their tailbone (in Dutch ‘leun achterover en zit op je stuitje’). They get the explanation to sit a little slumped (in Dutch ‘zit daarvoor een beetje onderuitgezakt’). Then they should tighten the pelvic floor and feel the movement against the chair (in Dutch ‘span de bekkenbodem aan en voel de beweging ten opzichte van de stoel’).
