# Supplementary figures and images for: The feasibility and usability of a personal health record for patients with multiple sclerosis: a 2-year evaluation study
Source: Front Hum Neurosci. 2024 May 22;18:1379780. doi: 10.3389/fnhum.2024.1379780 (PMC11150701; doi:10.3389/fnhum.2024.1379780)

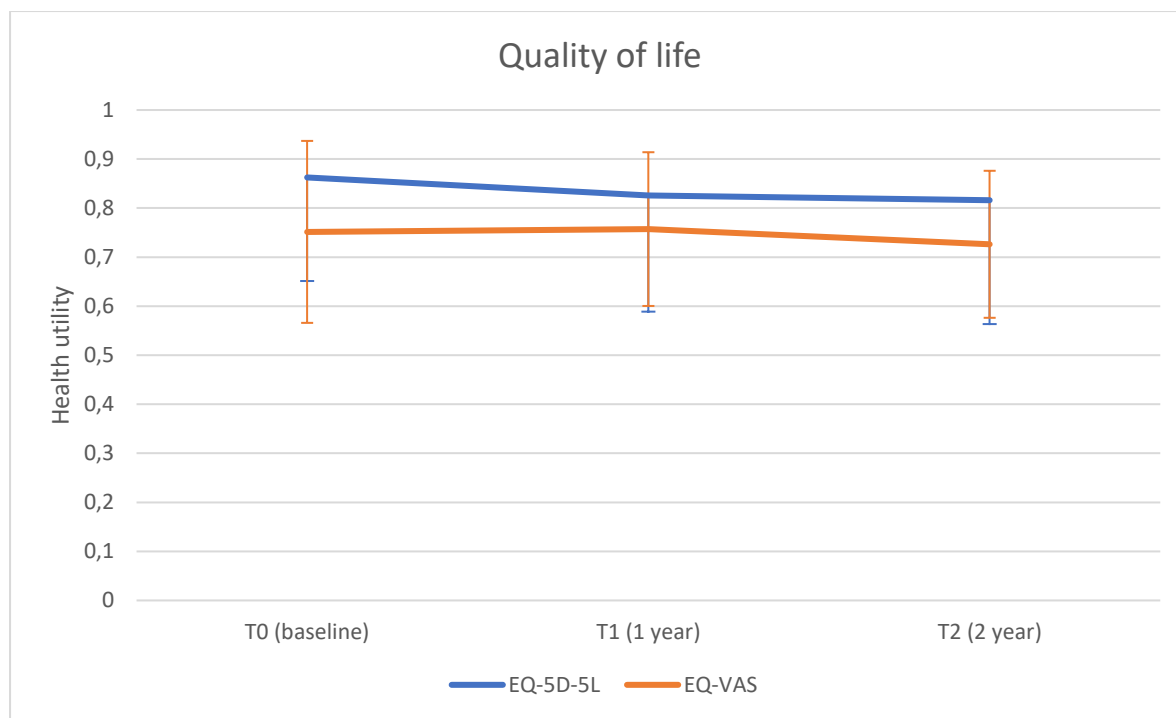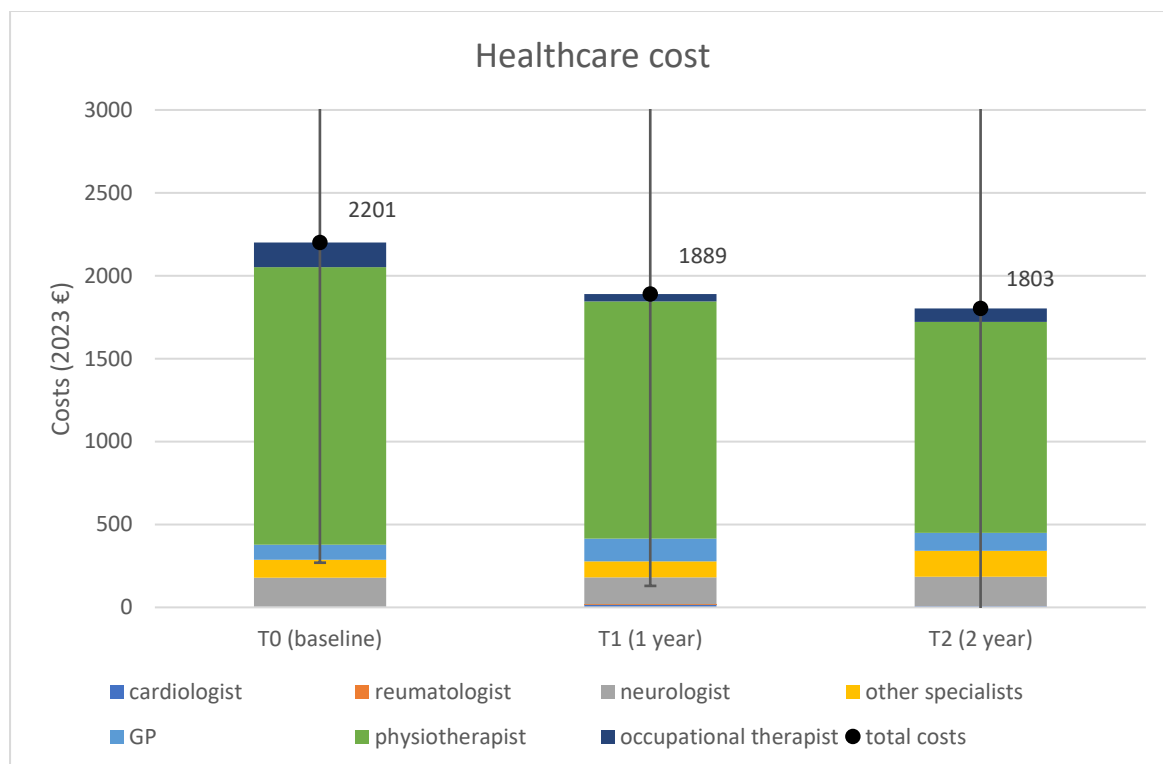

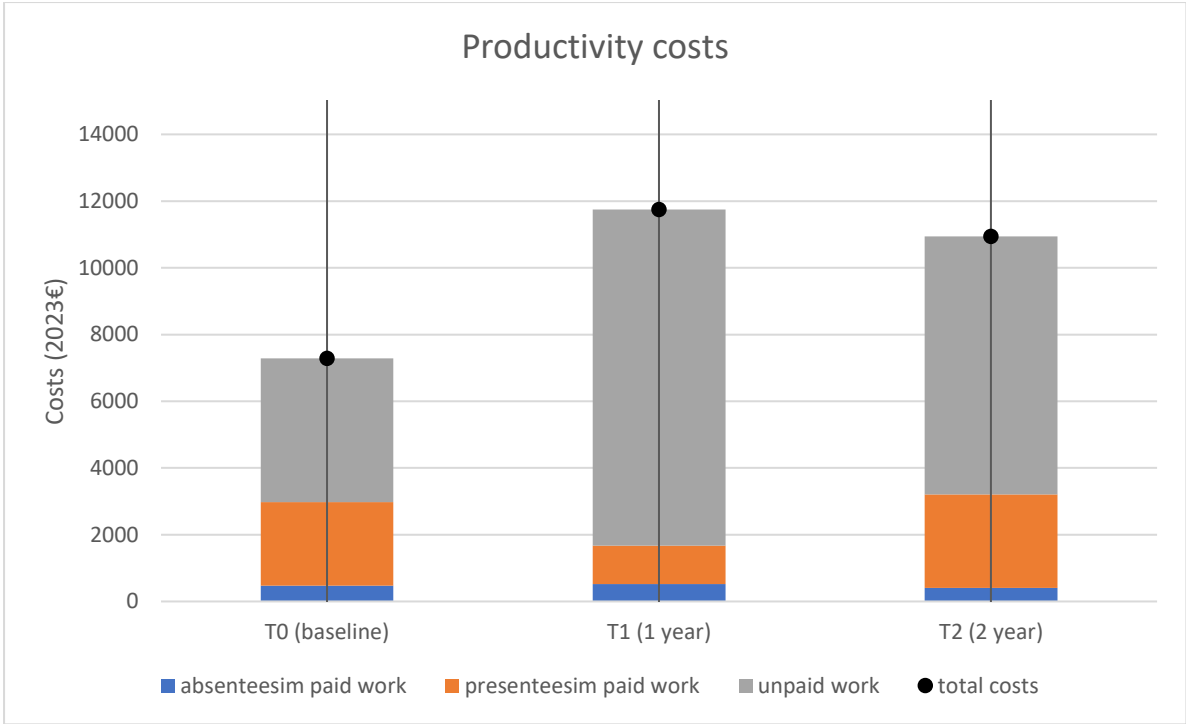

Supplement: Supplementary file 2 [file Image_1.PDF]
